# Evaluation of Biodegradable Alloy Fe30Mn0.6N in Rabbit Femur and Cartilage through Detecting Osteogenesis and Autophagy

**DOI:** 10.1155/2023/3626776

**Published:** 2023-01-18

**Authors:** Shimin Hao, Tianyu Yang, Ao Zhang, Penghao Wang, Hua Jiang, Dianlin Shen, Lei Guo, Mao Ye

**Affiliations:** ^1^Department of Orthopedic Surgery, First Affiliated Hospital, China Medical University, Shenyang, Liaoning 110001, China; ^2^Department of Orthopedic Surgery, Chaoyang Central Hospital, Chaoyang, Liaoning 122000, China; ^3^Department of Nursing, First Affiliated Hospital, China Medical University, Shenyang, Liaoning 110001, China

## Abstract

Biodegradable iron alloy implants have become one of the most ideal possible candidates because of their biocompatibility and comprehensive mechanical properties. Iron alloy's impact on chondrocytes is still unknown, though. This investigation looked at the biocompatibility and degradation of the Fe30Mn0.6N alloy as well as how it affected bone formation and chondrocyte autophagy. In vivo implantation of Fe30Mn0.6N and Ti6Al4V rods into rabbit femoral cartilage and femoral shaft was carried out to evaluate the degradation of the alloy and the cartilage and bone response at different intervals. After 8 weeks of implantation, the cross-sectional area of the Fe30Mn0.6N alloys lowered by 50.79 ± 9.59%. More Ca and P element deposition was found on the surface Fe30Mn0.6N rods by using energy dispersive spectroscopy (EDS) and scanning electron microscopy (*P* < 0.05). After 2, 4, and 8 weeks of implantation, no evident inflammatory infiltration was seen in peri-implant cartilage and bone tissue of Fe30Mn0.6N and Ti6Al4V alloys. Also, implantation of Fe30Mn0.6N alloy promoted autophagy in cartilage by detecting expression of LC3-II compared with Ti6Al4V after implantation (*P* < 0.05). Fe30Mn0.6N alloy also stimulated early osteogenesis at the peri-implant interface compared with Ti6Al4V after implantation (*P* < 0.05). In the in vitro test, we found that low concentrations of Fe30Mn0.6N extracts had no influence on cell viability. 15% and 30% extracts of Fe30Mn0.6N could upregulate autophagy compared to the control group by detecting beclin-1, LC3, Atg3, and P62 on the basis of WB and IHC (*P* < 0.05). Also, the PI3K-AKT-mTOR signaling pathway mediated in the upregulation of autophagy of chondrocytes resulting in exposure to extract of Fe30Mn0.6N alloy. It is concluded that Fe30Mn0.6N showed degradability and biocompatibility in vivo and upregulated autophagy activity in chondrocytes.

## 1. Introduction

Recently, iron-based metals have attracted much concern due to their potential biodegradability and excellent mechanical properties for use as bone implant materials [1]. Other metals such as titanium alloys, stainless steel, and cobalt alloys have already been used as implants. Nevertheless, these metals cannot match the elastic modulus of bones and cause stress shielding, leading to delaying or even impeding the fracture healing process [2]. Magnesium alloy has been researched in many studies and showed little prospect for clinic use due to its fast corrosion rate, limited load capacity, and local tissue reaction associated with hydrogen generation [3]. Moreover, pure iron also failed clinically because of its low degradation rate [4, 5]. Iron-based alloys have shown a proper degradation rate in some studies. Some researchers have explored the use of iron-based alloys as biodegradable vascular stents [6]. However, its use as an orthopedic implant material has not been clarified yet. The novel Fe30Mn0.6N alloy has an austenite (c phase) structure alloyed with manganese, which can reduce the magnetic susceptibility and exhibit compatibility with magnetic resonance imaging (MRI) [7]. The right proportion of manganese can change the degradation rate of the alloy [8]. The addition of N element can make the alloy have better corrosion resistance and mechanical properties [9]. In previous studies on Fe-Mn alloy in vivo, bones were mostly selected as objectives to evaluate alloy's degradation and its effects on adjacent osteocytes. In some cases, however, the implants may be close to cartilage, and degradation products of the implants can disperse into the articular cavity in some intra-articular fractures. We aim to study the effects of Fe30Mn0.6N on cartilage through in vivo experiment. Autophagy is a normal physical process for cellular survival involved in engulfing damaged organelles and proteins by autophagosomes when cells are under some stimuli such as starvation, hypoxia, and oxidative stress. Chondrocytes are the only cellular constituents of cartilage. Previous research has demonstrated that enhanced autophagy in chondrocytes can inhibit the onset of osteoarthritis, cell aging, and death by influencing intracellular metabolism. Furthermore, autophagy plays a pivotal role in protecting cells by removing reactive oxygen species (ROS) [10]. Therefore, it is very important to study the effects of the implants on cartilage autophagy. LC3-II was selected as the marker for autophagy in in vivo experiment. The PI3K/AKT/mTOR signaling pathway plays a significant role in downregulating autophagy [11]. In this study, we also explored whether the PI3K/AKT/mTOR signaling pathway was involved in the regulation of autophagy caused by the implants in the in vitro experiment.

## 2. Methods

### 2.1. Materials and Extract Preparation

**Figure 1:**
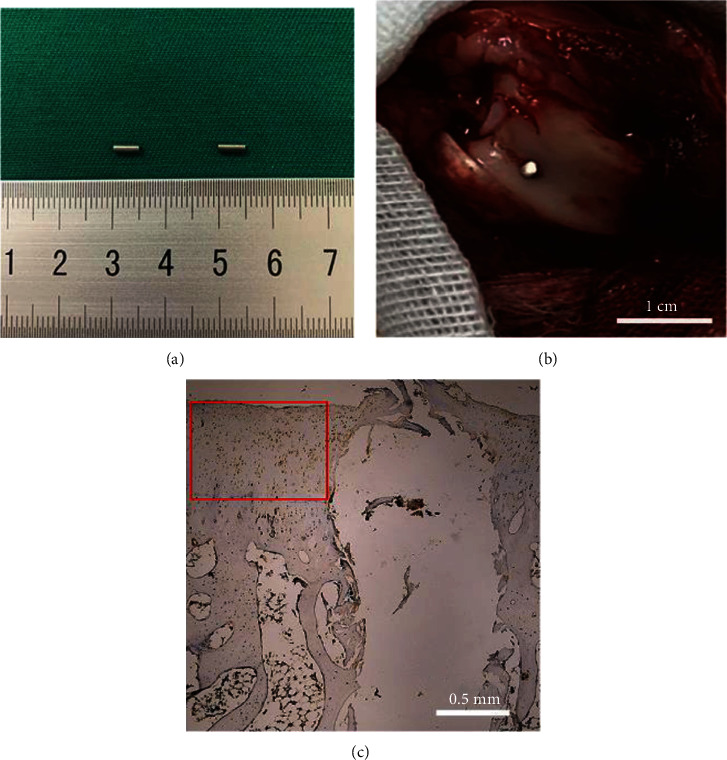
(a) Fe30Mn0.6N rod (left) and Ti6Al4V rod (right). (b) Implantation of rods in distal femoral articular cartilage and subchondral bone tissue of rabbit knees. The white line represents 1 cm. (c) The blank region was where the rod was implanted; adjacent regions marked by red square were selected for observation. The white line represents 0.5 mm.

The iron-based alloy, Fe30Mn0.6N, and Ti6Al4V alloy were offered by the Institute of metal research, Chinese Academy of Sciences, Shenyang, China. The element constituents are given in Table 1. For the purpose of the experiment, the alloy was processed into rod and disc forms. The discs in the in vitro experiments were processed from extruded Fe30Mn0.6N rods with a length of 2.0 mm and a diameter of 10 mm. In the in vivo experiment, Fe30Mn0.6N alloy and Ti6Al4V alloy were processed into rod samples with a diameter of 1.0 mm and a length of 5.0 mm (Figure 1(a)). Alloy samples were ground with SiC papers of 1000 grits, followed by ultrasonic cleaning in alcohol and drying in warm dry air. Ti6Al4V alloy samples were prepared in the same way. Then, the Fe30Mn0.6N rods and the Ti6Al4V alloy rods were implanted in the left and right condyles of rabbit knee joints, respectively (Figure 1(b)). Alloy extracts were created in compliance with ISO 10993-5, an international standard. The alloy disc samples were soaked for 24 hours in 10% fetal bovine serum (FBS, Hyclone) and Dulbecco's modified Eagle medium-high glucose (DMEM, Gibco™, Invitrogen). The surface area to extraction medium ratio was 1.25 cm^2^/ml, and the incubator's settings were 37°C and 5% CO_2_. After extracting the medium, the supernatant was taken out, centrifuged, and kept at 4°C. The 100% extract was diluted to 15%, 30%, and 45% using DMEM in order to examine the impact of extract concentration on the autophagy of the chondrocytes.

### 2.2. Evaluation of Biocompatibility and Degradability of the Alloys In Vivo

#### 2.2.1. Animals and Surgery

All animal experiments were conducted in accordance with the ISO 10993-2:1992 animal welfare requirements. New Zealand rabbits (license number: SCXK (hu) 2008-0016) were used in the in vivo experiment. The animal tests were performed after getting animal ethics approval from the Institutional Animal Ethics Committee, China Medical University, according to “Principles of laboratory animal care” (Approval No. CMU2019295 dated December 6, 2019). The study was conducted using 70 adult male New Zealand white rabbits weighing 1.2 ± 0.5 kg. Before surgery, the rabbits were required to adapt to the environment. The animals were housed in cages for ten days, with humidity and temperature maintained at about 60% to 70% and 22°C. They had free access to fresh water and were subjected to alternate 12-hour cycles of light and darkness.

In the in vivo experiment, anesthesia was performed on all rabbits with 0.5% pentobarbital sodium solution prior to implant surgery. A 1 mm hole was drilled, followed by the implantation of one Fe30Mn0.6N rod into the left femoral condyle of the animal. A Ti6Al4V rod was implanted into the right femoral condyle in the same way as shown in Figure 1(b). Similarly, Fe30Mn0.6N and Ti6Al4V rods were inserted into the left and right femoral shafts of the rabbits, respectively. After the operation, all rabbits were injected with gentamycin subcutaneously to prevent infection. Following surgery, the rabbits were given free range of motion in their cages without assistance from the outside. At 2, 4, and 8 weeks following surgery, 5 rabbits in the Fe30Mn0.6N alloy group and the Ti6Al4V alloy group were euthanized, respectively.

#### 2.2.2. Hematoxylin and Eosin (HE) and Immunohistochemical Analysis

For the cartilage group, 40 experimental rabbits were sacrificed before implantation, 2, 4, and 8 weeks after implantation, respectively. The condyle specimens, including the implants, were removed in order to observe biodegradation. The specimens were then fixed in 4.0% formaldehyde solution, decalcified in 15% EDTA solution for three weeks, and then embedded in paraffin. Following this procedure, the leftover implants were delicately removed from the condyles with a needle, leaving a hole at the location of implantation in the condyle samples. Then, the condyle specimens were embedded in paraffin for the following staining and observation. Each specimen was consecutively cut into 5 mm thick slices. As shown in Figure 1(c), the blank rectangular region is where the alloy was implanted and the adjacent region was used to finish HE and immunohistochemistry staining for further observation. Hematoxylin and eosin (HE) stains were used to stain deparaffinized slices before they were microscopically analyzed. In order to detect LC3-II, immunohistochemistry staining was performed. Deparaffinized sections were pretreated with 0.3% H_2_O_2_ for 30 min at room temperature to block endogenous peroxidase activity. This was followed by three washes with distilled water. The slices were then processed in a 10% solution of regular goat serum for 40 min at room temperature. The sections were coated with primary antibodies (Bioss, China), diluted in 0.1 M PBS (1 : 200), and then kept at 4°C for 48 hours in a humid environment. The sections were then incubated with the secondary antibody (biotin-goat anti-rabbit IgG, Boster, China), diluted in 0.1 M PBS (1 : 200), for 20 min at 37°C, after three rounds of washing with 0.1 M PBS for a total of 6 min. The slices were washed with PBS four times for a total of 20 minutes, then incubated in 0.03% 3,3′-diaminobenzidine (DAB; Boster, China) for five minutes before being counterstained with hematoxylin. Positive proteins showed brown staining.

Similarly, 30 experimental rabbits were sacrificed 2, 4, and 8 weeks after implantation, respectively, to get femoral shafts with implants. Following a fixation step in a 4.0% formaldehyde solution, the samples underwent 4 weeks of decalcification in a 15% EDTA solution. The connection between the remaining alloy implants and bone became loose during this process, and the alloy rods were carefully removed by a needle. The sections were then embedded with paraffin. Following HE staining, antibodies for BMP-2 were employed for immunohistochemistry staining on the tissues.

On an Olympus BX50 microscope, routine examination and immunohistochemistry were shown. The mean optical density (MOD) value of the implant/bone interface was calculated in order to quantitatively evaluate the immunohistochemistry. Five fields were randomly selected for quantitative evaluation for each specimen and factor at the implant/bone and implant/cartilage interface of each slice, and the MOD values were computed using software ImageJ.

#### 2.2.3. Evaluation of Degradation of the Alloy In Vivo

The Fe30Mn0.6N alloy and Ti6Al4V alloy implanted for 2, 4, and 8 weeks were examined under a scanning electron microscope (Hitachi, Japan) to observe alloy degradation and assess the corrosion of the alloy. Energy dispersive spectroscopy was used to measure the surface's chemical composition (Oxford, United Kingdom). The weight percentages (wt) of calcium (Ca) and phosphorus (P) were examined using GraphPad Prism software, using Ti6A14V alloys as the control group. Then, epoxy glue was used to embed the inserted rods. As a control, epoxy resin was also used to embed unimplanted Fe30Mn0.6N and Ti6Al4V alloy specimens. To obtain the alloy implant cross-sections, the samples were then processed with emery paper. Software called ImageJ was used to measure the leftover implant area.

### 2.3. In Vitro Evaluation of Autophagy in Chondrocytes

#### 2.3.1. Cell Culture

In the in vitro tests, rabbit chondrocytes (CP-Rb002, Cmbio, Shanghai, China) were grown in a solution with 100 g/ml streptomycin, 100 U/ml penicillin, and complete DMEM with 10% fetal bovine serum. The chondrocytes were grown in a 37°C, 5% CO_2_ cell incubator. The condition of the cells' growth was regularly monitored, and the cell culture media were changed every two days. When subcultured, rabbit chondrocytes expanded to a confluence level of more than 80%.

#### 2.3.2. Cell Viability Assay

The Cell Counting Kit-8 (CCK-8) was used to determine cell viability in accordance with the manufacturer's recommendations. In a 96-well plate, rabbit chondrocytes were seeded with 10% FBS at a density of 8 × 10^3^ cells per well. Chondrocytes were then exposed to Fe30Mn0.6N alloy extracts at several concentrations (0% used as the control, 15%, 30%, and 45%) and 100% Ti6Al4V alloy extracts for 24 hours. Each well received a dose of CCK-8 reagent solution (Dojindo, Kumamoto, Japan) (1 : 10). A wavelength of 450 nm was used to measure the optical density (OD) values.

#### 2.3.3. Transmission Electron Microscopy

The chondrocytes were collected after 24 hours of incubation in 30% Fe30Mn0.6N alloy extract and 100% Ti6Al4V alloy extract by performing centrifugation at 1500 rpm at 4°C for 5 minutes. Cells were washed with PBS three times after the supernatant was removed. The cells were then put under a fixative solution of 2.5% glutaraldehyde in phosphate buffer (0.01 M, pH = 7.4). The cells were first cleaned with phosphate buffer, fixed in 1% osmic acid at 4°C for 1.5 hours, and then dehydrated in a graduated series of acetone. Embedding in 60% epoxy resin followed, then waiting until it hardens. Utilizing an ultramicrotome, very thin slices were produced. Lead citrate and uranyl acetate were used to stain the sections. Images of cells were seen through a TEM. The autophagic vesicles in each cell were counted, and then, 10 fields of view were selected for each sample randomly.

#### 2.3.4. Western Blot

A six-well plate was utilized for cell culture, 2 × 10^6^ rabbit chondrocytes were added to each well, and Fe30Mn0.6N alloy extract of different concentrations (0%, 15%, 30%, and 45%) was used to treat the cells for 24 hours. After the cells were collected, they were washed with cold PBS and lysed with ice followed by cytocentrifugation (4°C, 12,000 × g). The supernatants were separated. The total protein concentrations were determined by the BCA Protein Assay Kit (Beyotime, Jiangsu, China). After being mixed with loading buffer, they were boiled for 10 min. The protein samples were separated by SDS-PAGE electrophoresis for 3 h and then transferred to the PVDF membranes. The samples were incubated with the primary antibody at 4°C overnight. The membrane was incubated with a goat anti-rabbit IgG peroxidase secondary antibody (Beyotime, Jiangsu, China) for 1 h; then, the image was captured using the ECL kit (ECL, NCM Biotech, Xiamen, China) and chemiluminescence system (Bio-Rad, USA). The primary antibodies were used as follows: anti-LC3B (Beyotime, Jiangsu, China), anti-beclin-1 (Beyotime, Jiangsu, China), anti-P62 (Abcam, Cambridge, UK), anti-Atg3 (Abcam, Cambridge, UK), and *β*-actin (Abcam, Cambridge, UK). Furthermore, 30% Fe-30Mn-0.6N alloy extract was used to study the signaling pathway by detecting relative proteins. The cells were first incubated in the alloy extract for 24 hours, then followed by the same procedures as before. The primary antibodies were used as follows: anti-AKT (Cell Signaling Technology, USA), anti-phosphor-AKT (Ser473) (Cell Signaling Technology, USA), anti-mTOR (Cell Signaling Technology, USA), anti-phosphor-mTOR (Ser2448) (Cell Signaling Technology, USA), and anti-LC3B and LY294002 (Beyotime, Biotechnology, China). ImageJ software was used to calculate the gray value.

#### 2.3.5. LC3 Immunofluorescence

First, Fe30Mn0.6N alloy extract was applied to rabbit chondrocytes for 24 hours at a 30% concentration. Sections were then fixed with 4% formaldehyde (Solarbio, Beijing, China), blocked with 5% BSA for one hour, treated with a rabbit monoclonal anti-LC3B antibody (Beyotime, Jiangsu, China) overnight, and then incubated with a goat anti-rabbit IgG peroxidase secondary antibody (Beyotime, Jiangsu, China). The samples were then counterstained with DAPI. The Zeiss LSM880 confocal microscope was utilized to visualize the LC3. Using ImageJ, images were analyzed, and their quantification was performed.

### 2.4. Statistical Analysis

The experimental results were expressed as mean values ± standard deviation (SD). The *t*-test and one-way analysis of variance were used to evaluate statistical significance. Data was gathered and examined using GraphPad prism. *P* < 0.05 was used to indicate statistical significance.

## 3. Results

### 3.1. Biodegradation of Fe30Mn0.6N Alloy Implant

**Figure 2:**
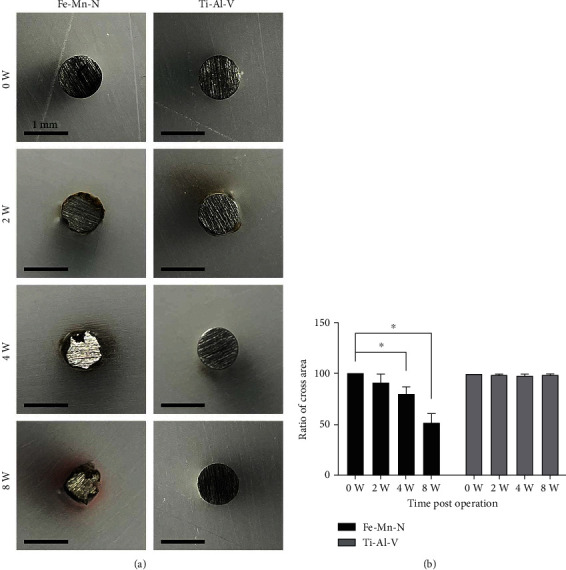
(a) Cross-sectional images of Fe-30Mn-0.6N alloy and Ti-6Al-4V alloy before implantation and 2, 4, and 8 weeks after implantation. The alloys before implantation were used as control groups. (b) Ratio of the residual cross-section area to the original cross-section area of Fe-Mn-N and Ti-Al-V alloy implant after different implantation times; ^∗^*P* < 0.05, *n* = 5.

**Figure 3:**
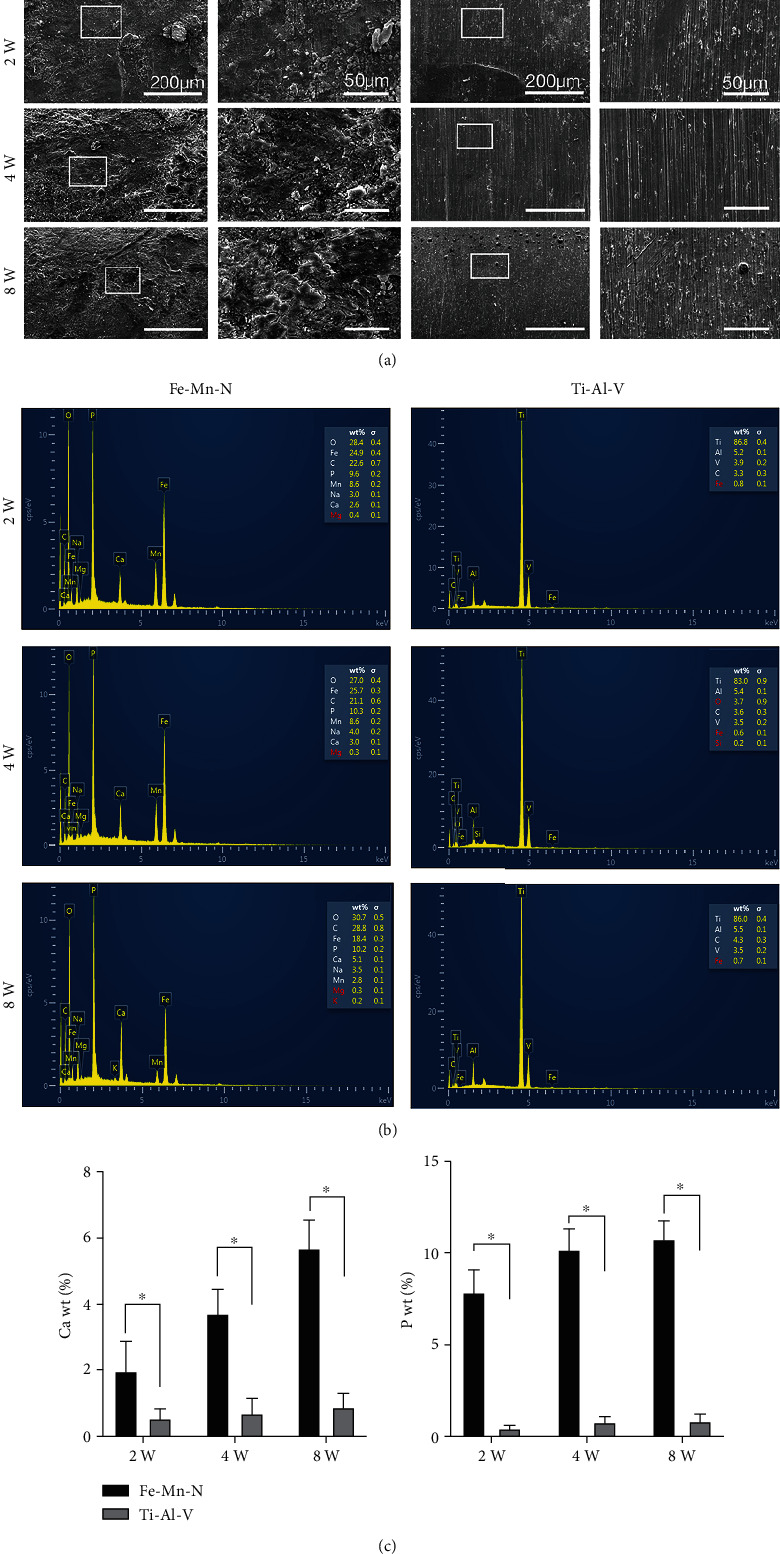
(a) Surface morphology of the alloy by SEM. White rectangle: selected the area for high magnification. (b) EDS analysis of elemental composition on superficial area of alloys. EDS spectrum of the area in (a). (c) Mean percentage of Ca and P present in alloys with EDS analysis. ^∗^*P* < 0.05, *n* = 6.

Shown in Figure 2(a) is the cross-section of Fe30Mn0.6N rods and Ti6Al4V rods before implantation and at 0, 2, 4, and 8 weeks postoperation, respectively. At week 2, the outline of Fe30Mn0.6N rod was no longer round, and the edge was like moth-eaten. Besides, there was some brown and black matter on the edge, which was due to the formation of iron oxides. At week 4, the outline was much rougher, and the residual area became smaller. At week 8 after implantation, the outline of Fe30Mn0.6N was even rougher than that of week 4. However, the outline and the shape of all Ti6Al4V alloy rods showed no obvious change and no deposition of black and brown matter on their edges.  Figure 2(b) illustrates the change of the residual cross-sectional area of the alloy implants at different times after operation; Ti6Al4V alloy showed no obvious change while Fe30Mn0.6N showed a decreasing tendency with time. At weeks 4 and 8, a significant difference in the residual area of the implants in the two groups could be seen. Scanning electron microscopy was used to investigate the implant surfaces. As shown in Figure 3(a), at week 2, the surface of the Fe30Mn0.6N alloy showed thin cracks and deposition of some small granular matter. At weeks 4 and 8, cracks and corrosion became more obvious. The surfaces of Ti6Al4V alloy were relatively smooth. No obvious cracks and corrosion were observed.  Figure 3(b) illustrates the EDS results of the square area on both Fe30Mn0.6N alloy and Ti6Al4V alloy after implantation. The results indicated that there was more P and Ca elemental deposition on Fe30Mn0.6N alloy's surface at 2, 4, and 8 weeks after the operation (*P* < 0.05) (Figures 3(b) and 3(c)).

### 3.2. Implantation of the Fe30Mn0.6N Alloy Led to a Hardly Detectable Inflammatory Response in Chondrocytes In Vivo

**Figure 4:**
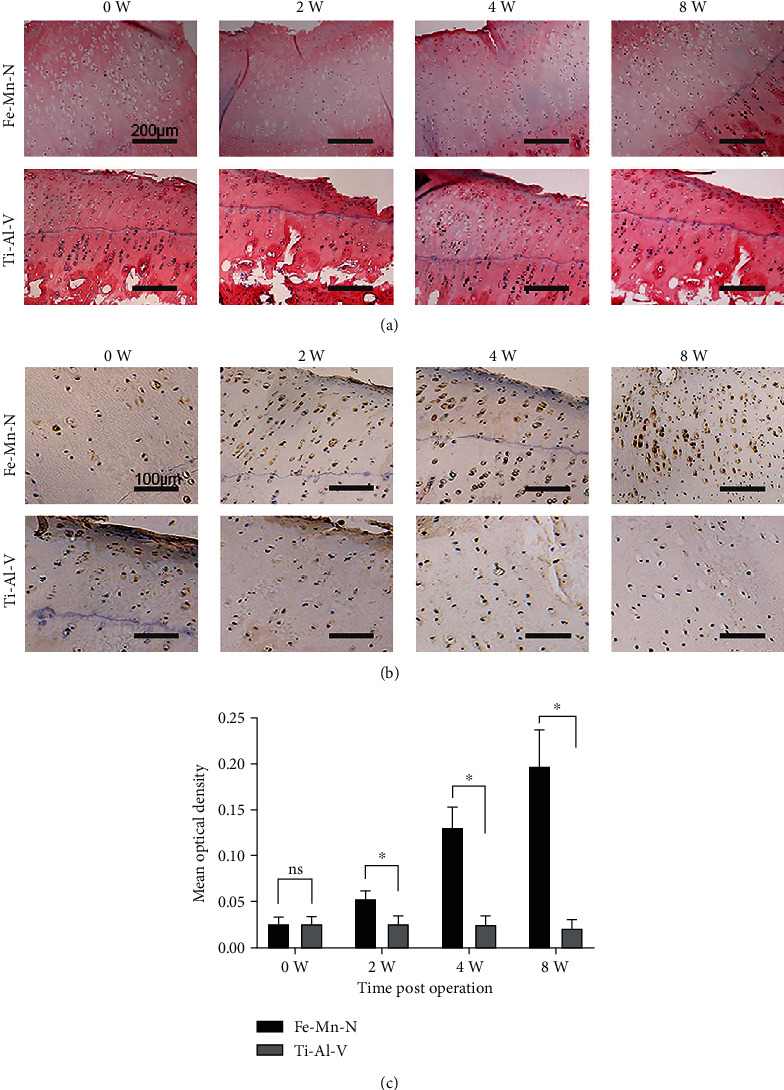
(a) The results of HE staining of cartilage before alloy implantation and 2, 4, and 8 weeks after implantation. (b) The IHC results of LC3-II in cartilage in the Fe-30Mn-0.6N alloy group and the Ti-6Al-4V alloy group before implantation and 2 and 4 weeks after implantation. (c) Analysis results of MOD of LC3-II expression in the Fe-30Mn-0.6N alloy group and the Ti-6Al-4V alloy group. ^∗^*P* < 0.05, *n* = 5.

Before and 2, 4, and 8 weeks after implantation, the animals were euthanized. The rabbits grew healthily and experienced no unexpected deaths throughout this time.  Figure 4(a) shows the HE-stained cartilage near the implanted rods. Neither the implantation of Fe30Mn0.6N alloy rods nor that of Ti6Al4V alloy rods caused obvious damage to the cartilage of the condyles. Cartilage in both the Fe30Mn0.6N group and the Ti6Al4V group showed normal morphology. Chondrocytes and the cartilage lacuna can be clearly found with complete normal shapes. No obvious inflammatory infiltration of neutrophils and monocytes was found on both groups.

### 3.3. Fe30Mn0.6N Alloy Implantation Did Not Inhibit Osteogenic Process In Vivo

**Figure 5:**
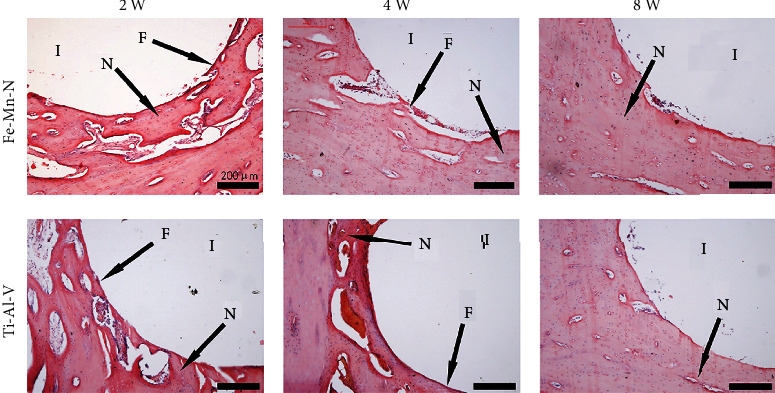
HE staining photographs of implant/bone interfaces after 2, 4, and 8 weeks postimplantation. I: implant; F: fibroblast band; N: newly formed osteoid tissue.

**Figure 6:**
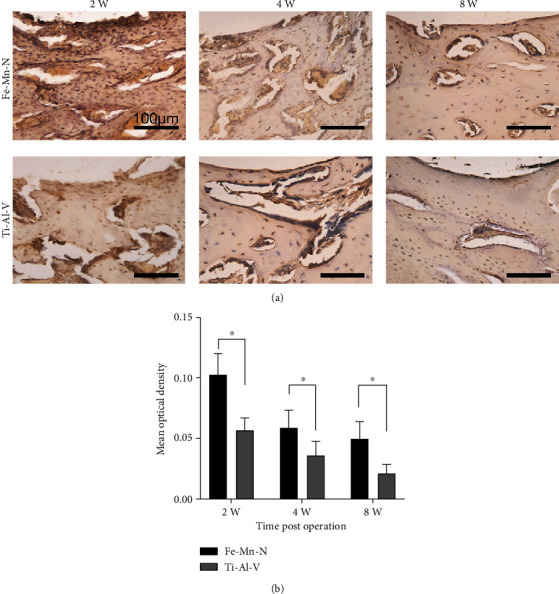
(a) Photomicrographs of BMP-2 expression at the interfaces between the implants and bones after 2, 4, and 8 weeks of implantation. (b) Analysis results of MOD of BMP-2 expression in Fe-30Mn-0.6N alloy group and Ti-6Al-4V alloy group. ^∗^*P* < 0.05, *n* = 5.

Figure 5 illustrates the microstructure of the interfaces between alloy implants and newly formed bone stained by HE at weeks 2, 4, and 8. For the Fe30Mn0.6N alloy, there was a continuous fibrous band forming on the interface between the implant area and the bone at week 2. And no obvious lymphocytic infiltration around the implant area was observed. The newly formed bone tissue was irregular and arranged relatively, as marked by “N.” At week 4, the fibrous band became thinner and the tissue around the implants became more compact and regular. After 8 weeks of implantation, the area around the implant was covered by newborn bone and bone trabeculae, while lymphocytic infiltration was not observed. For the Ti6Al4V alloy, the fibrous band could be observed, but it was not continuous at week 2. After 4 weeks postimplantation, the newly formed bone became more compact and a fibrous layer could be observed. After 8 weeks postimplantation, bone trabeculae became more compact.  Figure 6(a) shows BMP-2 staining at the adjacent area near implants after different periods.  Figure 6(b) shows the MOD values of BMP-2 expression after different periods of implantation. At week 2, positive activities were observed near the implants on both Ti6Al4V alloy and Fe30Mn0.6N alloy groups. At weeks 4 and 8, newly developed osteoid tissues were seen at the border, and BMP-2 expression on both groups gradually reduced. The Fe30Mn0.6N alloy group shows a higher MOD value than the Ti6Al4V alloy group in the expression of BMP-2 at each interval after implantation. A significant difference could be found at each interval (^∗^*P* < 0.05, *n* = 5).

### 3.4. Fe30Mn0.6N Extracts of Low Concentration Did Not Inhibit Viability of Chondrocytes In Vitro

**Figure 7:**
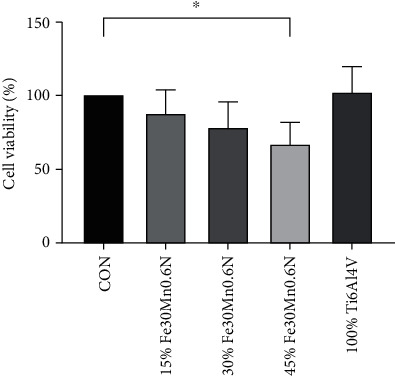
The CCK-8 assay was carried out to measure cell viability. ^∗^*P* < 0.05 versus control group. Values represent means ± SD (*n* = 5).

Rabbit chondrocytes were processed with different concentrations of Fe30Mn0.6N extracts (0, 15%, 30%, and 45%) and 100% Ti6Al4V extract for 24 h. The CCK-8 assays were used to evaluate cell viability. As shown in Figure 7, no significant differences were found between the control group and 15% and 30% Fe30Mn0.6N extracts. The viability of chondrocytes decreased in 45% Fe30Mn0.6N extract (*P* < 0.05). 30% extract was chosen for further research. There were no significant differences between the control group and 100% Ti6Al4V extract.

### 3.5. Fe30Mn0.6N Alloy Induced Upregulation of Autophagy in Chondrocytes In Vivo

Immunohistochemistry was used to evaluate autophagy by examining the expression of LC3-II in cartilages (Figure 4(b)). At each time point following surgery, the Fe30Mn0.6N implant displayed a greater MOD value in the LC3-II expression than the Ti6Al4V implant, and a significant difference was found between the Fe30Mn0.6N implant and Ti6Al4V group (*P* < 0.05) at 2, 4, and 8 weeks postoperation, but the MOD value had no significant difference in two groups before implantation. And the expression of LC3-II in the Fe30Mn0.6N group exhibited an increasing tendency with time.

### 3.6. Fe30Mn0.6N Alloy Induced the Autophagy in Chondrocytes In Vitro

**Figure 8:**
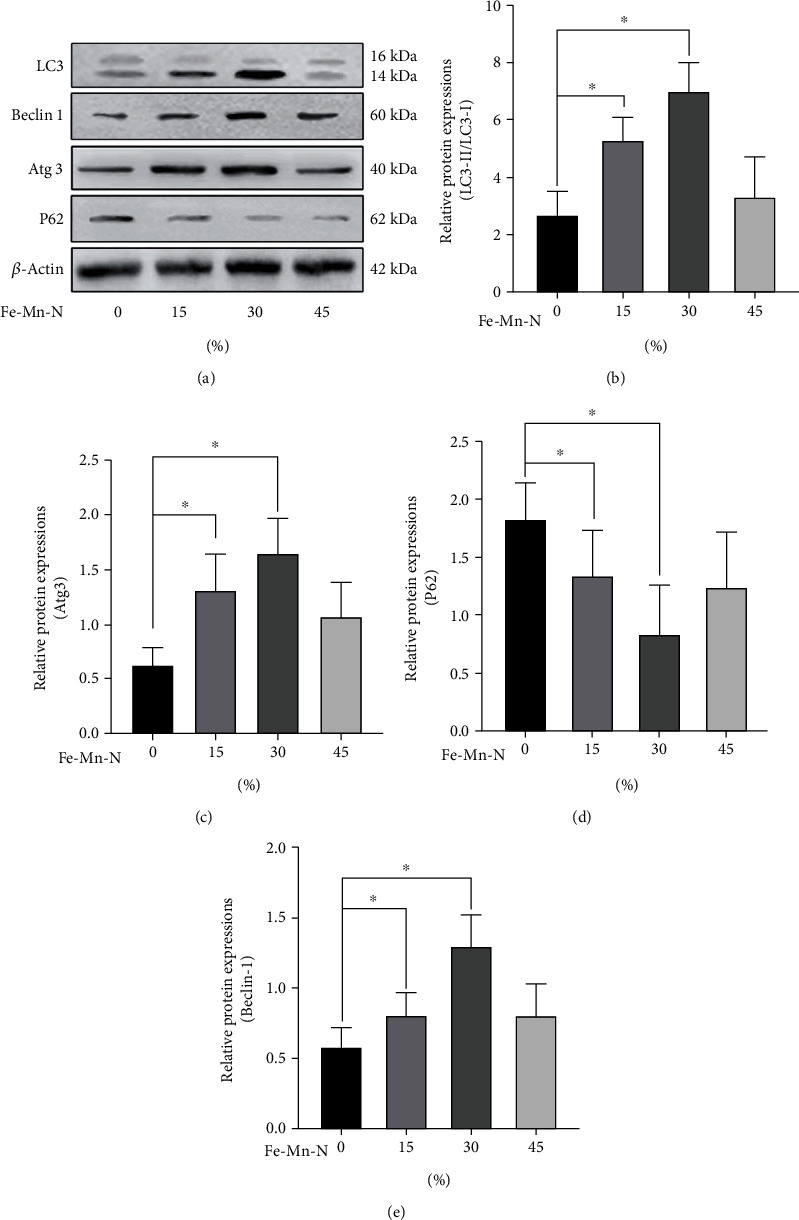
The results of the Western blot: (a) representative experiment depicting relative expression of protein in chondrocytes treated with 0%, 15%, 30%, and 45% Fe30Mn0.6N alloy extracts; (b–e) analysis of Western blot test results of LC3-II/LC3-I, ATG3, P62, and beclin-1, ^∗^*P* < 0.05, *n* = 6. Data was presented as the mean ± SE.

**Figure 9:**
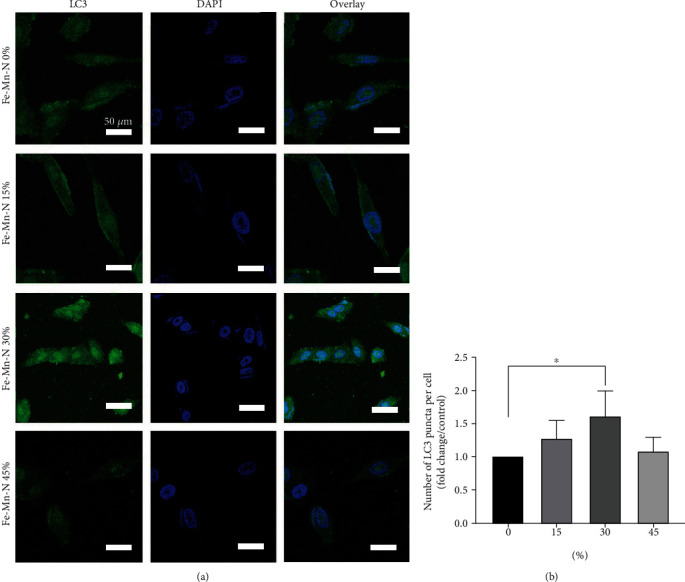
The results of the fluorescence microscopy analysis: (a) representative immunofluorescence images depicting LC3-II in chondrocytes treated with Fe30Mn0.6N alloy extracts of 0%, 15%, 30%, and 45% for 24 h; the white line represented 20 *μ*m in images; white bar = 50 *μ*m; (b) the graphs represented the quantification of LC3 puncta average number in chondrocyte using ImageJ quantification tool (^∗^*P* < 0.05). Quantification was performed from 3 experiments with >25 cells counted for each condition.

**Figure 10:**
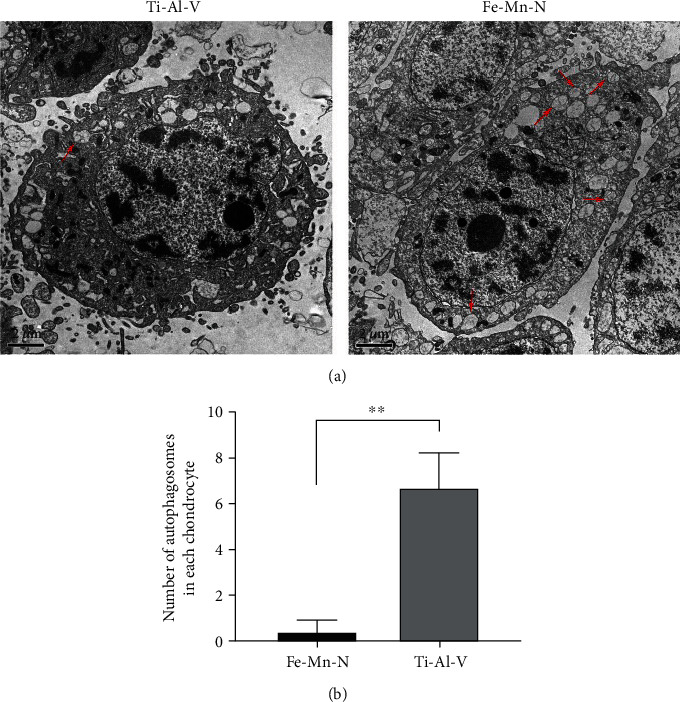
Fe-Mn-N alloy extract promoted chondrocyte autophagy. (a) Images of bilayer autophagic bodies in chondrocytes observed by TEM; the red arrows in images represented autophagic bodies of cells. (b) Number of autophagic bodies of chondrocytes. ^∗∗^*P* < 0.01, *n* = 3.

As shown in Figure 8, when the concentration of the extract was below 30%, the conversion of LC3-I to LC3-II, beclin-1, and Atg3 was positively related to the concentration and then decreased when the concentration reached 45%. On the other hand, P62 first decreased until the concentration went up to 30% and then increased. Immunofluorescence of LC3-II also showed a similar trend as shown in Figure 9. When treated with 30% concentration extract, the puncta in each chondrocyte were more than in other groups. The 30% extract of Fe30Mn0.6N was selected to further study its effects on autophagy by using TEM. As shown in Figure 10, chondrocytes in both groups under TEM appeared with normal complete structures. Autophagic vesicles with two layers in the cytoplasm could be found. Compared with the Ti6Al4V alloy group, Fe30Mn0.6N groups had a higher amount of autophagic vesicles (*P* < 0.01), reflecting that autophagy was more active in the Fe30Mn0.6N group.

### 3.7. PI3K-AKT-mTOR Signaling Pathway Mediated the Regulation of Chondrocytes

**Figure 11:**
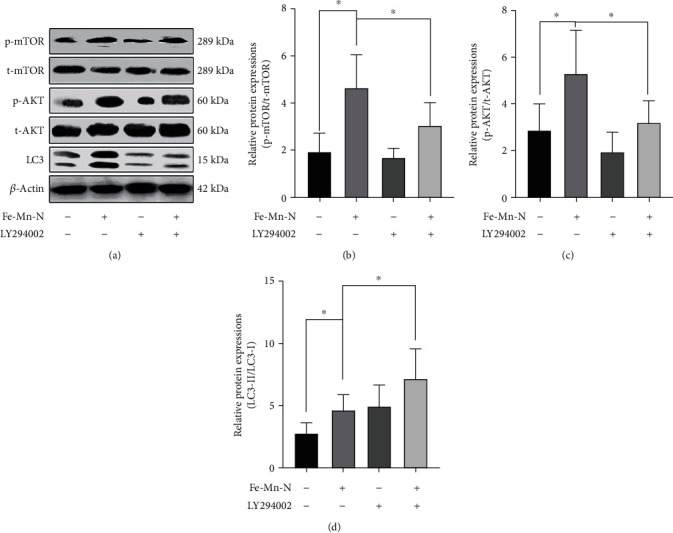
Fe-30Mn-0.6N extract and LY294002 treatment enhanced autophagy by inhibiting PI3K/AKT/mTOR signaling pathway. Cell lysates were collected to analyze the cytoplasmic expression of AKT and mTOR phosphorylation and LC3-II/I expression by Western blot with actin as a reference. The bars indicate the relative value compared with the control group (^∗^*P* < 0.05, *n* = 6).

To further study whether changes of autophagy in chondrocytes were related to the PI3K-AKT-mTOR signaling pathway, we used LY294002, a PI3K specific inhibitor, to pretreat the cells for 1 h at a concentration of 25 mM. The total protein was collected after incubation with 30% Fe30Mn0.6N extract for 12 h. In chondrocytes cultivated in 30% Fe30Mn0.6N alloy extract, the expression of proteins linked to the PI3K/AKT/mTOR signaling pathway was determined using Western blot. The samples were designed as blank control, Fe30Mn0.6N alloy extract, LY294002, and Fe30Mn0.6N alloy extract+LY294002. Western blot results showed that the expression levels of P-AKT and P-mTOR were significantly decreased after LY294002 exposure (*P* < 0.05; Figures 11(b) and 11(c)), while the LC3-II/I ratio increased (*P* < 0.05; Figure 11(d)). These results demonstrated that autophagy activities of chondrocytes after Fe30Mn0.6N extract exposure are negatively correlated with PI3K/AKT/mTOR signaling pathway activity at low concentrations of the extracts.

## 4. Discussion

Corrosion at different stages in vivo was demonstrated in the study, and degradation products were also found at the periphery of the implant. The residual Fe30Mn0.6N rod showed a brown and black zone on the edge, which is due to the formation of Fe_2_O_3_ and Fe_3_O_4_, respectively. On the implants, erosion was also seen using SEM. According to the EDS data, P, Ca, C, and O made up the majority of the deposition. In light of previous research, we assume that the formation of deposition was due to the production of calcium phosphates and metal oxides [7, 12].

Some research also demonstrated that iron in Fe-Mn alloy transformed to ferrous ion by anodic reaction [7]. These ferrous ions may be absorbed by adjacent chondrocytes and synovial cells. Iron is one of the essential micronutrients in the human body. Excessive iron exists in cells by the synthesis of ferritin, an iron storage protein [13]. Extracellular ferric ions first bind to transferrin, making it soluble and nontoxic. Then, loaded transferrin reacts with specific receptors on the cellular surface, followed by endocytosis [14]. Many studies have shown that an overload of iron could result in various diseases by depositing in different systems such as liver cirrhosis and osteoporosis [15, 16]. Besides, iron overload could induce the upregulation of apoptosis and autophagy. Also, iron overload promoted ROS production [17–20].

However, no obvious and visible degradation products of Mn or N were observed in this study. It has been reported that Mn ion is the main corrosion product of Mn metal. However, the distribution and concentration of products of Mn in vivo still needed to be studied further. Manganese, an essential nutrient for cells, plays important roles in varieties of biochemistry reactions as cofactors of some enzymes such as arginase and glutamine synthetase (GS) and pyruvate carboxylase [21, 22]. Some evidence has proved that long-term overexposure to manganese could lead to neurological and cardiovascular dysfunction [23]. No evidence of toxic reaction was found in experimental animals. Further study will be conducted to reveal the systemic distribution and concentration of manganese during the degradation process. To alter the alloy's mechanical characteristics, nitrogen is frequently added. According to some reports, adding the right quantity of N to alloys can increase their elongation and proof stress [9, 24, 25]. Also, some coronary stents made from nitrogen-containing stainless steel have been tested adequately in the in vivo and in vitro experiments and showed good biocompatibility [26, 27]. In the in vivo experiment, no inflammatory cell infiltration was observed near the implanted area at each interval after implantation, which was probably due to the slow releasing rate of degradation, because faster release is more likely to trigger severe inflammation. The results of this study indicated that partial degradation took place at 8 weeks on the Fe30Mn0.6N rod and that the Fe alloy may function as a mild irritant locally. Hendra's study on Fe-Mn alloys demonstrated that Fe-Mn alloy has different characteristics and different influence on the fibroblast cells in vitro from those of single element metal because iron and manganese atoms form a stable face-centered cubic crystal structure, also known as C phase [28]. Thus, Fe-Mn alloy shows less toxic and inhibition effects than using either the single element or the simple mixture of iron and manganese without forming an alloy in vitro degradation test.

The upkeep of chondrocyte homeostasis is greatly aided by autophagy, for the substances digested by autophagy need to be replenished by newly synthesized macromolecules. Also, autophagy can repair the damage to cartilage cells and inhibit the occurrence and development of OA [29]. Existing studies have suggested that beclin-1 combines with type III phosphatidylinositol enzyme (PI3K) to form complexes for autophagosome. On the other hand, LC3 is responsible for the elongation and maturation of phagosomes [30]. To evaluate biocompatibility and safety, we also studied its influences on autophagy of chondrocytes.

It has been reported that excessive iron storage can stimulate cells to produce ROS and enhance apoptosis [31]. Nevertheless, the growth of cartilage and bone in the in vivo experiment was not inhibited. ROS could impair cellular homeostasis, leading to oxidative stress and mitochondrial dysfunction [32, 33]. In this process, oxidative stress could enhance autophagy, which, in turn, may act to decrease oxidative damage by engulfing and removing oxidized substances. According to the results of the measure of positive rate for LC3-II, autophagy activity in Fe30Mn0.6N groups was higher than that in Ti6Al4V alloy groups. We assumed that Fe30Mn0.6N released ferrous ions and they induced cytoprotective autophagy by removing ROS. The growth of cartilage and bone was not curbed, which may contribute to the following factors. Firstly, the degradation rate of Fe30Mn0.6N alloy was relatively slow, and there is no excessive accumulation of degradation products especially ferrous ion and manganese ion. Moreover, degradation products could be diluted and removed by body fluid. What is more, autophagy functioned to protect the chondrocytes by removing ROS. The kinase mammalian target of rapamycin (mTOR), which is controlled by a variety of stimuli including hypoxia and hunger, is a crucial regulator of the autophagic process [11, 34]. The PI3K/AKT pathway, upstream of mTOR, suppresses autophagy response by activating mTOR. Based on a previous study, we supposed that PI3K/AKT signaling was related to the regulation of autophagy. Further study showed that the PI3K-AKT-mTOR signaling pathway was involved in the upregulation of autophagy of chondrocytes due to the exposure to extract of Fe30Mn0.6N alloy.

## 5. Conclusion

In this study, the new iron-based alloy, Fe30Mn0.6N alloy, showed no toxic effects on laboratory animals after 2, 4, and 8 weeks of implantation in vivo. The new alloy also showed proper degradation characteristics, and the growth of cartilage and bone was not badly influenced after the implantation of the alloy. The implantation of Fe30Mn0.6N alloy also upregulated autophagy, functioning as a cytoprotective factor. Further studies in vitro showed that Fe30Mn0.6N alloy extracts could increase autophagy activity at low concentrations of extracts and the PI3K-AKT-mTOR pathway is involved in the regulation of autophagy. This research provided a new perspective for the biocompatibility evaluation of alloy implantation. In general, Fe30Mn0.6N alloy showed both biocompatibility and degradability in this study, and we provided some evidence for its future use as a biodegradable material.

## Figures and Tables

**Table 1:** The chemical element composition of Fe30Mn0.6N.

Materials	Chemical compositions (wt %)						
	Mn	N	C	O	S	P	Fe
Fe30Mn0.6N	29.32	0.58	0.015	0.01	0.016	0.007	Balance

## Data Availability

The data used to support the findings of this study are available from the corresponding authors upon request.
